# The Redox Proteome*

**DOI:** 10.1074/jbc.R113.464131

**Published:** 2013-07-16

**Authors:** Young-Mi Go, Dean P. Jones

**Affiliations:** From the Division of Pulmonary, Allergy, and Critical Care Medicine, Department of Medicine, Emory University, Atlanta, Georgia 30322

**Keywords:** Glutathione, Redox Regulation, Systems Biology, Thiol, Thioredoxin

## Abstract

The redox proteome consists of reversible and irreversible covalent modifications that link redox metabolism to biologic structure and function. These modifications, especially of Cys, function at the molecular level in protein folding and maturation, catalytic activity, signaling, and macromolecular interactions and at the macroscopic level in control of secretion and cell shape. Interaction of the redox proteome with redox-active chemicals is central to macromolecular structure, regulation, and signaling during the life cycle and has a central role in the tolerance and adaptability to diet and environmental challenges.

## Introduction

Redox biology is the study of oxidation-reduction processes associated with life. Early 20th century research on O_2_ as an electron acceptor linked electron transfer reactions to generation of ATP (1, 2). In 1955, research with ^18^O_2_ and mass spectrometry (MS) showed that atoms from O_2_ were introduced into biomolecules (3, 4), stimulating research on mechanisms of O_2_ activation (5, 6). This research was focused mostly on enzyme cofactors rather than the redox activity of the translated proteome.

At the same time, protein aggregation and oxidative inactivation introduced artifacts in protein chemistry and enzymology (7). Removal of contaminating trace metals, O_2_, and/or inclusion of GSH or dithiothreitol protected against these processes. The susceptibility of protein to oxidation contributed to the study of oxidative damage in biologic systems, termed oxidative stress (8, 9). The discovery of superoxide dismutase (10) fueled this research and the related study of lipid peroxidation, oxidative DNA damage, and oxidative protein chemistry. Advances in cancer biology and molecular biology led to discovery of NADPH oxidases, enzymes that produce reactive oxygen species (superoxide and H_2_O_2_) for redox signaling (11). At the same time, advances in MS and crystallography supported detailed studies of the proteome.

**FIGURE 1.**
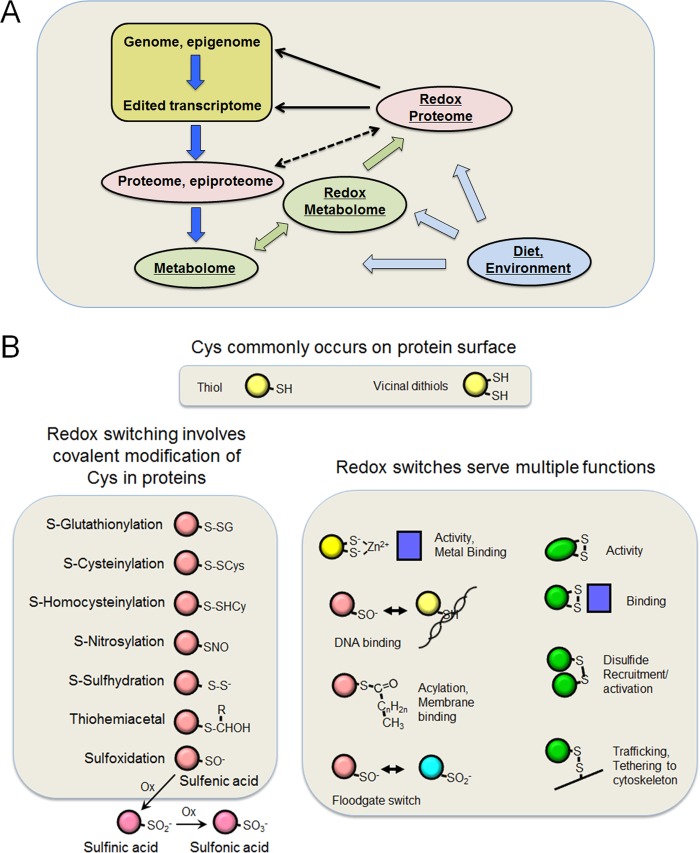
**Redox proteome as the interface of the epiproteome, diet, and environment.** *A*, the redox proteome is a subset of post-translational modifications of protein considered here as the epiproteome (indicated by the *dashed arrow*). This is considered in analogy to the epigenome and genome as a system to provide function and regulation beyond that of the translated sequence of amino acids. The redox proteome includes amino acids that undergo reversible redox reactions (Cys, Met, and Sec) and others that are irreversibly modified by reactive species in oxidative stress (Lys, Trp, and others). The redox proteome impacts the genome and epigenome through altered DNA and RNA binding and altered trafficking, activities, and structures of associated proteins. Metabolic activities, especially in cell signaling, impact the metabolome. The redox metabolome is a subset of the metabolome that alters structure and function of the redox proteome. NADPH/NADP, GSH/GSSG and Cys/cystine are central components of the redox metabolome. The metabolome and redox proteome respond to diet and environmental influences (*blue arrows*) to protect against oxidant stress and other environmental challenges. *B*, covalent modifications of Cys provide a versatile structure-function switching system. Cys commonly exists on the surface of proteins either alone (monothiol) or in close proximity to another Cys residues (vicinal dithiols) (*top*). The reactivities are impacted by vicinal cationic amino acids, which enhance ionization and make Cys more reactive. Vicinal dithiols tend to form disulfides upon oxidation, whereas monothiols undergo reversible oxidation to sulfenic acid and mixed disulfides with low molecular mass thiol chemicals, GSH, Cys, and homocysteine (*HCy*; *left*, *inside box*). Under highly oxidizing conditions, sulfenic acid is further oxidized to sulfinic and sulfonic acids (*left*, *below box*). Other modifications also occur, such as *S*-nitrosylation, *S*-sulfhydration, and thiohemiacetal formation. These changes, as well as Zn^2+^ binding and acylation, can result in altered protein-protein interactions, DNA or RNA binding, or membrane interaction (*right*).

In this minireview, we provide an overview of redox components of the translated proteome that connect electron transfer to biologic structure and function. We do not include the central enzymology of oxidoreductases, *e.g.* oxidases, hemoproteins, NAD-linked intermediary metabolism, or mitochondrial electron transfer. We focus instead on reversible reactions of the Cys proteome, which has an inherent tendency to undergo oxidative post-translational modification under aerobic conditions. Irreversible covalent modifications provide a complementary system for prolonged biologic redox signaling and have been addressed elsewhere (12). Both are relevant to oxidative stress and redox signaling and, through interactions with the metabolome, provide an interface with diet and environmental exposures (Fig. 1*A*). Disruption of these mechanisms contributes to human disease.

## Definition of Redox Proteomics

“Redox proteome” is a collective term for components of the proteome that undergo reversible redox reactions and those modified irreversibly by reactive species during oxidative stress. Only three of the amino acids undergo reversible reaction (Cys, Met, and selenocysteine (Sec)^2^) but many amino acids (*e.g.* Trp, Tyr, and Arg) and the peptide backbone react with products generated during oxidative stress (13). The redox proteome interacts with the redox metabolome, a redox-active subset of the metabolome, with NADPH/NADP^+^, GSH/GSSG, and cysteine/cystine being specifically relevant to the redox proteome. The metabolome includes low molecular mass biochemicals generated by intermediary metabolism and chemicals from the diet, microbiome, pharmaceuticals, commercial products, and environment (14). Many are linked to NADH/NAD and other redox systems and can indirectly impact the redox proteome.

The redox proteome impacts the genome and epigenome, RNA processing and translation, and other post-translational modifications of the translated proteome (Fig. 1*A*). In analogy to the epigenome and genome, the epiproteome includes the translated proteome with covalent modifications to provide function and regulation beyond that of the translated sequence of amino acids. As a component of the epiproteome, the redox proteome is controlled primarily by endogenous metabolism but responds to chemicals from the diet, air, and other exposures. These redox reactions have a central role in integrating homeostatic, adaptive, and defense responses between the epigenome, epiproteome, and external environment.

### 

#### 

##### Redox Steady State

**FIGURE 2.**
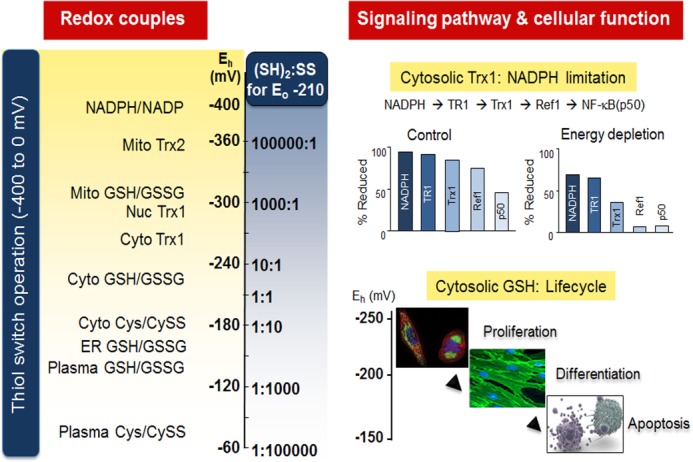
**Non-equilibrium steady states of redox couples direct metabolism, structure, and macromolecular trafficking.** Central thiol/disulfide couples (*left*) serve as redox hubs and include small molecules of the redox metabolome and redox-active proteins, such as Trx1 and Trx2. These exist with a range of steady-state redox potentials (*E_h_*, calculated with the Nernst equation (see text)) spanning nearly 400 mV from mitochondrial NADPH/NADP to plasma Cys/cystine (*CySS*). The difference between redox couples (Δ*E_h_*) is proportional to −Δ*G*, describing the energetics of electron transfer. The ratio of (SH)_2_ to SS for a dithiol/disulfide couple with *E_o_* = −210 mV illustrates the relative abundance of the forms if the protein is equilibrated at the respective *E_h_* value. *Upper right*, kinetic limitation is shown for proteins in the pathway from NADPH to NF-κB (p50) under both control and energy-limiting conditions in cultured cells. *Lower right*, the *E_h_* of cytosolic GSH/GSSG becomes progressively oxidized in the life cycle of cells from proliferation to differentiation to apoptosis. Such change could, in principle, impact proteins via glutaredoxin-dependent *S*-glutathionylation (30) or reflect changes in H_2_O_2_ generation and GSH peroxidase activity. *Mito*, mitochondria; *nuc*, nuclear; *cyto*, cytosolic; *ER*, endoplasmic reticulum; *TR1*, Trx reductase 1. Cell images are from Invitrogen and the Genetics Home Reference (http://ghr.nlm.nih.gov).

Thioredoxin (Trx) and GSH provide effective thiol antioxidant systems, yet ongoing oxidation maintains a non-equilibrium steady-state oxidation of protein Cys in cells. This steady-state oxidation is not fully understood because non-enzymatic protein thiol oxidation with physiologic oxidants is relatively slow (15), as illustrated by redox-sensitive green fluorescent protein, which requires minutes to oxidize even with added H_2_O_2_ (16). However, measurement of multiple proteins in a redox pathway in cells (Fig. 2) shows that reduction is kinetically limited (17). Because the rates of oxidation and reduction of individual thiols in cells are difficult to measure, redox effects are often quantified in terms of the steady-state redox potential (*E_h_*). *E_h_* is calculated using the Nernst equation: *E_h_* = *E_o_* + *RT*/*nF* ln[oxidized molecule]/[reduced molecule], where *R* is the gas constant, *T* is the absolute temperature, *n* is the number of electrons transferred, and *F* is Faraday's constant. The NADPH/NADP couple is maintained at about −400 mV in cells and serves as the principal reductant to reverse oxidation by O_2_ (O_2_/H_2_O at approximately +600 mV). The displacement of thiol/disulfide systems from equilibrium is illustrated by GSH/GSSG (Fig. 2), with intermediate *E_h_* in plasma (−138 mV) (18) and in liver (−255 mV) (19). Similarly, Trx1 has an intermediate value (−270 mV) (20).

##### Reversible Redox Elements of the Proteome

The thiol of Cys is the most extensively characterized component of the redox proteome and is the main focus of this minireview. This does not imply that Cys is more important than Sec or Met. Sec is present in only 25 human proteins, but these include central redox enzymes, Trx reductases, and GSH peroxidases (21). The Met proteome is similar to the Cys proteome but is less studied. If one excludes Met for initiation of translation, the human genome encodes 174,000 Met residues (17). A small percentage exists in enzyme active sites, but others contribute to the three-dimensional structure and molecular interactions (22). About 5–10% of the total Met proteome is present *in vivo* as Met sulfoxide (MetO) (23). A family of MetO reductases (Msr) reduces peptidyl-MetO back to peptidyl-Met (24). Genetic manipulation of MsrA impacts longevity (25), suggesting the importance of peptidyl-MetO in sensitivity to oxidative stress but not excluding other redox functions of peptidyl-Met oxidation.

## Special Character of the Cys Proteome

Early research showed that about half of all enzymes are sensitive to thiol reagents, and studies of protein structure revealed contributions of disulfides to three-dimensional configuration (26) and to protein processing and trafficking (27). Additionally, x-ray crystallography and molecular biology revealed widespread Zn^2+^ binding to Cys in proteins (28, 29). The fundamental character of Cys as a “sulfur switch” (30), discussed below, was recognized only more recently.

### 

#### 

##### Oxidation of the Cys Proteome

The human Cys proteome includes 214,000 Cys residues encoded in the genome. Only the thiol form is translated due to the specificity of the tRNA^Cys^ synthetase, so all modified forms represent post-translational modifications. In cells and tissues, 5–12% of total protein Cys is oxidized, and this can be increased to >40% by adding oxidants. Oxidation occurs through 1e^−^ or 2e^−^ reactions, producing thiyl radicals or sulfenic acids and disulfides, respectively (Fig. 1*B*). Introduction of sulfenic acids is termed sulfenylation (31); this reaction and disulfide formation represent central components of the reversible redox proteome. The most commonly studied disulfide formation is *S*-glutathionylation (32), but *S*-cysteinylation and protein-protein disulfide formation also occurs. Successive oxidation produces sulfinic and sulfonic acids (Fig. 1*B*).

##### Other Modifications of the Cys Proteome

Peptidyl-Cys reacts with other biologic molecules to support complementary mechanisms, such as *S*-nitrosylation and *S*-sulfhydration (Fig. 1*B*). Inclusion of such modifications into systems biology models of the redox proteome requires a multidimensional description because some specific peptidyl-Cys residues can undergo different modifications, *i.e.* a specific Cys may be glutathionylated, cysteinylated, nitrosylated, or sulfhydrated (33–37). *S*-Nitrosylation of proteins also occurs (38), and *S*-nitroso groups can be transferred between protein thiols (39). Sulfhydration of protein thiols by H_2_S also occurs (40, 41) and could support a system of sulfane transfer (42) analogous to nitrosyl transfer. How multiple modifications are integrated within the proteome remains uncertain because global proteomic analysis showed overlap between protein Cys undergoing *S*-nitrosylation and sulfenylation, but the extent of this overlap is limited (43). Reversible reaction with aldehydes, including monosaccharides, to form thiohemiacetals is also possible, but the extent and biologic functions of such modifications are not known. Thiol modifications that result from post-translational modifications of the proteome by electrophiles are termed the adductome (44). These include a biochemical signature of environmental exposure to reactive chemicals (44) and also of endogenously generated reactive lipids and lipid products from radical reactions of oxidative stress that can function in signaling (12).

## Sulfur Switches

**FIGURE 3.**
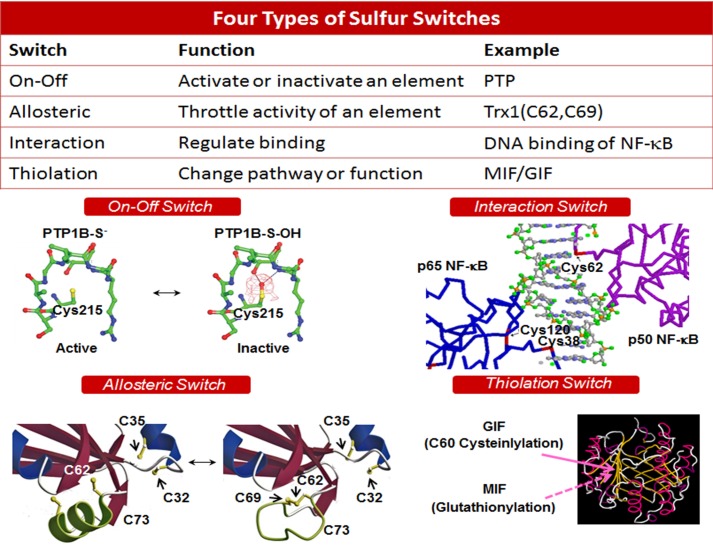
**Types of sulfur switches.** Sulfur switches can turn systems on and off, provide allosteric regulation, change the binding interactions, and/or have a chameleon-like effect on the character of a protein (*upper*). Crystal structures are provided to illustrate each. In PTP1B (*PTP*), oxidation at the active site Cys^215^ residue turns off activity (*middle left*) (45). Oxidation of Cys^38^ in NF-κB (p65) or Cys^62^ in NF-κB (p50) results in loss of DNA binding (*middle right*). Formation of a disulfide (Cys^62^–Cys^69^) in a surface α-helix in Trx1 (20) results in an allosteric-like decrease in interaction with Trx reductase (*lower left*). A switch in cysteinylation of Cys^60^ (Protein Data Bank) to glutathionylation results in a change from glycosylation-inhibiting factor (*GIF*) activity to macrophage migration inhibitory factor (*MIF*) activity (*lower right*).

Sulfur within the redox proteome has a fundamentally important use as a structure-function switch. At the molecular level, there is often no meaningful separation of structure and activity related to redox effects on sulfur. At the macroscopic level, however, structure and activity can be measured independently. Because of this, we list types of switches (Fig. 3) as a framework to consider redox links between metabolism, biologic structure, and activity.

### 

#### 

##### Types of Sulfur Switches

An “on-off” switch provides a qualitative activity change, such as inactivation of protein-tyrosine phosphatases by oxidation in kinase signaling (45). An allosteric switch adjusts activity, as occurs in regulation of enzymes, such as Trx1 (46). An interaction switch provides a change in binding character. Examples include oxidation of Cys in the transcription factor NF-κB, which prevents its binding to DNA (47), and palmitoylation/myristoylation of endothelial nitric-oxide synthase to enhance binding to membrane (48). Such changes activate complex formation in cell signaling (49) and sequential processing in protein maturation and secretion (50). Finally, different thiolation at a specific Cys residue can change the function of a protein. For example, a switch in cysteinylation of Cys^60^ to glutathionylation results in a change from glycosylation-inhibiting factor activity (51, 52) to macrophage migration inhibitory factor activity (53). Together, these sulfur switches provide diverse capabilities for integration of biologic structures and functions. These sulfur switching mechanisms of the redox proteome evolved with the genome to provide a common system to coordinate and optimize complex biologic systems.

##### Operation of Sulfur Switches

The reversible nature of thiol oxidation to sulfenate and disulfide makes the thiol a versatile element for switching structure and function. Schafer (30) defined sulfur switches in terms of stoichiometry of electron transfer involving 1e^−^ processes, such as glutathionylation, and 2e^−^ processes, such as disulfide bond formation. The activity of the former varies as a function of thiol/disulfide ratio, whereas that of the latter varies as a function of [GSH]^2^/[GSSG] (19). The latter is also expressed in terms of the corresponding *E_h_* to allow comparison with other redox-active systems. Several physiologic mechanisms involving protein glutathionylation occur (32), and hundreds of proteins are glutathionylated during oxidative stress (54). Oxidation of a dithiol (Cys^62^–Cys^69^) in a surface α-helix of human Trx1 provides an example of a 2e^−^ oxidation (20). The Keap1 control system for the transcription factor Nrf2 has 26 Cys residues and appears to have Cys residues undergoing both 1e^−^ and 2e^−^ oxidation (55, 56). Some active sites, *e.g.* kinase and phosphatases, are also considered to be sulfur switches because they are reversibly inactivated by oxidation.

##### Distinction of Redox Signaling and Redox Sensing

**FIGURE 4.**
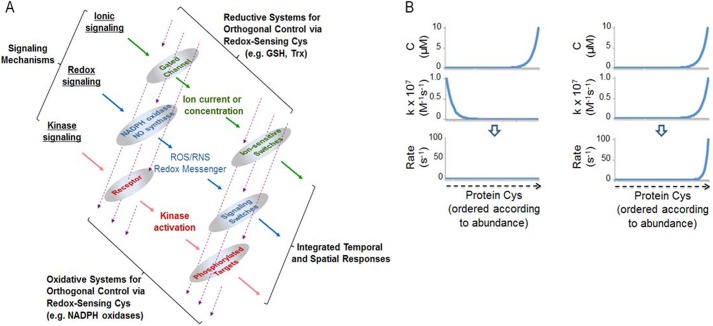
**Integrated function of the redox proteome.** *A*, redox-sensing Cys residues provide an orthogonal control system to regulate and integrate biologic systems without impacting mechanisms. Redox-signaling mechanisms are presented as pathways (*diagonal right arrows*) that serve to signal cell stress and other responses. These include controlled H_2_O_2_ production by regulated NADPH oxidases and redox-signaling Cys. They function in parallel with other signaling pathways, such as kinase signaling and ion-gated signaling (*diagonal right arrows*). These are viewed as providing relatively rapid and transient signals. Redox-sensing Cys residues integrate these signaling pathways without impacting their mechanisms (*diagonal left arrows*). Although poorly defined experimentally, the latter can be conceived as a background poise of the cellular H_2_O_2_ generation by mitochondria, Nox4, and other oxidases (*lower left*) and opposed by reductant systems dependent upon Trx and GSH (*upper right*). These have slow kinetics relative to signaling mechanisms and thereby provide a more long-term phenotypic control. This allows a single signaling mechanism to be used effectively in different cell types and states of cell division, growth, differentiation, or apoptosis. These signaling and sensing Cys switches operate within the non-equilibrium states maintained by the central redox hubs (Fig. 2) and together provide a versatile system for integrated spatial and temporal control of cell structure and function. *ROS*/*RNS*, reactive oxygen and nitrogen species. *B*, broad ranges of protein thiol reactivity and abundance are known to occur in the redox proteome. These provide a redox proteomic structure to support localized redox signaling within an inherently stable redox system. *Upper left panel*, protein abundance (*C*) is given on the *y axis* from femtomolar to 10 μm, expressed as a function of each respective protein, listed in order of abundance on the *x axis. Middle left panel*, a hypothetical condition is shown in which the second-order rate constant (*k*) for protein thiol reaction with H_2_O_2_, from 1 to 10^7^ m^−1^ s^−1^ (73), is varied in opposition to abundance for the same proteins. *Lower left panel*, the product of the rate constant and concentration (*k*·*C*) for each protein under this condition shows that all proteins contribute equivalently to the rate of H_2_O_2_ metabolism. *Right*, the rate constant (*middle panel*) is varied in proportion to abundance (*upper panel*). The product (*k*·*C*) for this condition shows that the most abundant protein thiols contribute 14 orders of magnitude more to the rate of H_2_O_2_ metabolism than the least abundant protein thiols (*lower panel*). This comparison shows that opposing co-evolution of abundance and reactivity of specific Cys residues within the proteome can account for an inherent stability of the redox proteome while also having specialized subsets of peptidyl-Cys for redox sensing and redox signaling.

The concepts of sulfur switching were derived from studies of redox signaling, but most sulfur switches do not function in discrete signaling pathways as described for analogous kinase signaling. Redox-sensing Cys residues serve as sulfur switches to regulate and integrate biologic functions (57). By operating through distinct Cys residues, redox-sensing Cys residues provide orthogonal control (Fig. 4*A*) for redox signaling and other activities by changing structure and function without changing molecular mechanisms.

##### Sulfur Switches and Evolution of the Cys Proteome

Based upon comparison of the percentage of tRNA^Cys^ among all tRNAs (3.28%) and the percentage of Cys in proteins (<2.2%), there is an evolutionary selection pressure against Cys in the proteome (58). Despite this, the percentage of Cys has increased from ∼0.5% in prokaryotes to 2.2% in mammals, showing an increase in Cys with evolution of complexity (58). This includes increased content of conserved/evolved Cys content (57). With an evolutionary selection against Cys, this implies a function for the evolved Cys, such as more extensive redox-signaling and redox-sensing activities. Analyses of conserved Cys residues in mitochondrial proteins detected by MS showed that >90% of the Cys residues were conserved among vertebrates, whereas only ∼70% of other amino acids in the same proteins were conserved (59).

## Methods for Study of the Redox Proteome

Detailed methods for the study of the redox proteome are available in the protein chemistry literature. Biologic studies have often included global measurements, such as total protein thiol measured by colorimetric methods; immunoassays, such as redox Western blotting of reduced and oxidized forms of specific proteins; and MS-based assays, such as the redox isotope-coded affinity tag methods for thiols (59–61) or DAz-2 reagents for sulfenic acids (62). Global measurements of protein thiols during experimental challenge provide a basis to evaluate the abundance of switchable redox elements in the Cys proteome. For instance, analysis of the rate of autoxidation of thiols in isolated nuclei shows that ∼10% of protein thiols are oxidized in 1 h and another 10% autoxidize more slowly, over a period of several hours. Extrapolation from such data suggests that 20,000–40,000 peptidyl-Cys residues are redox-sensitive. These could function as redox sensors (57) as discussed above or as decoy thiols to protect critical active sites from oxidants and electrophiles (63).

### 

#### 

##### Redox Western Blot Analysis

Immunoassays are useful to measure oxidation of individual proteins in cell and tissue extracts (20, 64, 65). Common methods include alkylation of thiols for separation from other forms by electrophoresis, followed by Western blotting (66). Labeling with thiol reagents containing biotin allows selection and/or detection with streptavidin to enhance flexibility in assay. Applications show that oxidation of individual proteins differs among compartments and varies with physiologic challenge and life cycle (64, 65, 67). The methods are useful to detect changes but can be difficult to calibrate because of multiple thiols in proteins, nonlinear responses, and epitope changes with thiol oxidation or alkylation. This is often a method of choice because of the ability to specifically examine redox changes in most proteins with standard biochemical laboratory equipment.

##### Mass Spectrometry-based Redox Proteomics

Adaptation of differential labeling approaches to the evaluation of the redox proteome has become increasingly popular (60–62, 68, 69). As with immunoassays, labeling with thiol reagents containing biotin allows detection or selection of peptides according to thiol content. These are useful with both two-dimensional gel electrophoresis coupled with MS/MS and two-dimensional chromatography-MS/MS methods. Oxidation of up to 4000 specific peptidyl-Cys residues, or ∼2% of the entire mammalian proteome, can be measured in a single experiment. Such data are useful in identifying specific Cys residues in cells. Many proteins are partially oxidized even under normal physiologic conditions (59), and possible functions of these Cys residues can be inferred by examination of the positions in x-ray crystal structures. Extensive application of these methods to Alzheimer disease, Parkinson disease, and other neurodegenerative diseases, as well as other human diseases and animal and cell models, has established the value of these approaches in improving the mechanistic understanding of redox biology and human disease (70).

## Integrated Redox Function of the Cys Proteome

Integration of detailed information on large numbers of redox-sensitive Cys residues requires systems biology approaches (46) and systematic data on the reactivity of specific thiols, protein abundance, spatial distribution, and organization into redox networks. A current view is that the Cys proteome exists in a dynamic steady state with organization into a branching network structure in which oxidation of specific thiols by O_2_, H_2_O_2_, and other oxidants is balanced by reduction by NADPH through Trx- and/or GSH-dependent systems (71). The *E_h_* values of Trx1 and GSH/GSSG couples are different, and neither is equilibrated with NADPH/NADP. In analogy to the use of regulons in genetics to describe groups of operons controlled by common elements, intermediate hubs are termed redox regulons (71). For instance, a Trx1 redox regulon and a GSH redox regulon regulate different subsets of the Cys proteome. An opposing set of oxidative redox regulons, operating in multiple subcellular compartments with secondary hubs, provides a bilateral redox network structure to accommodate specific control of each of the 214,000 Cys residues in the proteome (71). In this bilateral design, NADPH is used both to support reductive redox regulons and to generate H_2_O_2_ to support oxidative redox regulons. This provides an inherent stability by linking rates of oxidation and reduction to a common precursor (72).

### 

#### 

##### Reactivity

The reactivity of peptidyl-Cys residues differs considerably according to the specific location within the three-dimensional structure of a protein. Measures of thiol reactivity with H_2_O_2_ show that rate constants differ by >6 orders of magnitude (73). This range of reactivity forces critical examination of the definition of network structure, as discussed above, in which multiple redox elements have variable reactivity with other elements (71). Many possible variations in circuitry can occur, and more complete definition of reactivities, as well as more complete measures of *in vivo* responses, will be needed to define the networks.

##### Abundance

Measures of absolute protein abundance are often not available, and lack of such data limits redox model development. The importance of abundance is apparent from the use of rate constants and abundance to calculate rate (Fig. 4*B*). Protein abundance varies by at least 7 orders of magnitude (74), and there are two systematic ways that this can be combined with rate constants to determine rates, either scaled proportionally or scaled inversely. Using the experimentally determined range for second-order rate constants (*k*) for protein thiol reaction with H_2_O_2_ (1–10^7^ m^−1^ s^−1^) (73), one can see that variation of the rate constant (Fig. 4*B*, *middle left panel*) in opposition to abundance (*C*; *upper left panel*) results in the product (*k*·*C*), such that all proteins have the same rate of H_2_O_2_ metabolism (*lower left panel*). If one considers variation of the rate constant (Fig. 4*B*, *middle right panel*) in proportion to abundance (*upper right panel*), the product shows that the most abundant protein thiols contribute 14 orders of magnitude more to H_2_O_2_ metabolism than the least abundant protein thiols (*lower right panel*). Thus, co-evolution of mechanisms to control abundance and reactivity can account for H_2_O_2_ signaling by a subset of proteins while at the same time having an overall system that is inherently stable to changes in H_2_O_2_ production.

##### Spatial Distribution

D'Autréaux (75) addressed spatial distribution of redox elements, which provides specificity in redox signaling. As they discussed, compartment- and microcompartment-specific control through local concentrations of oxidants and reductants may be a common biologic principle. Directional movement through the secretory pathway involves O_2_-driven oxidation of peptidyl-Cys by the endoplasmic reticulum oxidizing system, thiol/disulfide exchange by protein-disulfide isomerases, and other sequential modifications during protein processing (50). A similar oxidative system for protein import into mitochondria occurs (76). Changes in extracellular redox potential regulate intracellular metabolism, but also changes in intracellular metabolism affect redox poise in the extracellular compartment (77).

##### Partial Oxidation of Proteins under Normal Aerobic Conditions

Early research on glutathionylation of actin at Cys^374^ (78) and redox Western analysis of protein-disulfide isomerase (79) and Trx1 (20) showed partial oxidation under physiologic conditions. MS studies on yeast extended this to show that many peptidyl-Cys residues are oxidized under control conditions (69), and studies with *Escherichia coli* (60), cultured cells (43), developing *Caenorhabditis elegans* (80), mouse aortic endothelial cells (81), and human colon carcinoma HT29 cells (59) confirmed and extended this finding. Functional pathway analysis showed that peptidyl-Cys mapped to functional networks according to percent oxidation (59). Proteins in cell regulation and actin cytoskeletal proteins appeared as different functional modules (81). Tubulins and other cytoskeletal protein, as well as docking proteins, such as 14-3-3, heat shock proteins, and many heteronuclear ribonucleoproteins, associate into functional networks (59). Many of these Cys residues are highly conserved in vertebrate evolution, indicating that this chemomorphic redox switching system contributes to the increased percentage of Cys in the proteome with evolution of complexity.

##### Actin-associated Redox Proteome

Actin provides an example of the complexity of redox systems and also illustrates the importance of dynamic regulation in cell functions. Disulfide bond formation (Cys^285^–Cys^374^) inhibits depolymerization of filamentous actin in red blood cells of sickle cell disease (82). Oxidation of cytoskeletal Cys causes protein aggregates, membrane blebbing, and cell death in other cells (83). Actin and other membrane-associated proteins (integrin α4 and myosin) are targets of oxidation in human peripheral blood mononuclear cells (84), and oxidation of extracellular *E_h_* increases F-actin formation (59), regulating actin (Cys^257^ and Cys^285^), several actin-associated proteins, and integrins (81). Glutathionylation of Cys^374^ by integrin-stimulated reactive oxygen species is essential for F-actin formation in cell spreading (85). Trx1 (Cys^62^) interaction with actin controls actin dynamics and is essential for anti-apoptotic function (86). Trx reductase is responsible for actin denitrosylation in neutrophils for cytoskeletal control and integrin β2 function (87). The association of Ref-1 (redox factor-1) with actin in thyroid nuclei modulates proliferation and differentiation (88). Sulfiredoxin acts on hyperoxidized peroxiredoxins (Prxs) (89, 90) to regulate deglutathionylation of actin (91). Cyclophilin B interaction with actin improves Prx activity in synaptosomes (92), and cyclophilin A (Cys^161^) controls glutathionylation/deglutathionylation of GAPDH, cofilin, and Prx. The cell survival mediator serine/threonine kinase Akt also functions in redox control of actin (93, 94). The number and complexity of these interactions illustrate that sulfur switches evolved as a network of control elements to integrate structure and function.

## Interactions with the Metabolome

The study of redox metabolism predates redox proteomics especially in understanding the central hubs of the redox metabolome. NADH/NAD is central to catabolism and ATP production, whereas NADPH/NADP is central to anabolism and control of the redox proteome (95). These hubs are not in redox equilibrium with each other and are not in redox equilibrium with low molecular mass redox hubs (GSH/GSSG and cysteine/cystine) or with protein redox hubs (Trx1 and Trx2). Nonetheless, as shown in Fig. 2, these hubs provide a context to maintain organization of redox metabolism and function of the redox proteome in which directionality is maintained by a stable non-equilibrium state. Temporal and spatial changes in the hubs provide a system for polarity in growth, differentiation, and other functions.

The recent development of high resolution metabolomics using high resolution MS and advanced data extraction (96, 97) has provided the capability to measure >20,000 chemicals in biologic extracts. Application to metabolomics of mitochondria shows the effect of Trx2 overexpression on hundreds of metabolites (98). Integration of metabolomics with redox proteomics provides an approach to elucidate adverse and beneficial interactions of diet and environment through the redox proteome.

## Summary

The redox proteome serves as an interface between genome-directed biologic structure and functions and the environmental determinants of those structures and functions. As such, the redox proteome is a central focus in biomedical research. The Cys proteome is particularly important in that the thiol of Cys serves as a versatile sulfur switch to link redox chemistry with structure and function. Advances in understanding the network structure of the redox proteome and interactions with the metabolome can be expected to improve the mechanistic understanding of redox signaling and oxidative stress impacting many aspects of human health and disease.

## Footnotes

2 The abbreviations used are:

Sec: selenocysteine
Trx: thioredoxin
MetO: Met sulfoxide
Prx: peroxiredoxin.

## References

[1] Keilin D. (1966) The History of Cell Respiration and Cytochrome, Cambridge University Press, Cambridge

[2] Mitchell P. (1979) Keilin's respiratory chain concept and its chemiosmotic consequences. Science 206, 1148–1159 388618 10.1126/science.388618

[3] Mason H. S. Fowlks W. L. Peterson J. (1955) Oxygen transfer and electron transport by the phenolase complex. J. Am. Chem. Soc. 77, 2914–2915

[4] Hayaishi O. Katagiri M. Rothberg S. (1955) Mechanism of the pyrocatechase reaction. J. Am. Chem. Soc. 77, 5450–5451

[5] Kovaleva E. G. Lipscomb J. D. (2008) Versatility of biological non-heme Fe(II) centers in oxygen activation reactions. Nat. Chem. Biol. 4, 186–193 18277980 10.1038/nchembio.71 PMC2720164

[6] Waterman M. R. (2005) Professor Howard Mason and oxygen activation. Biochem. Biophys. Res. Commun. 338, 7–11 16153596 10.1016/j.bbrc.2005.08.120

[7] Ziegler D. M. (1985) Role of reversible oxidation-reduction of enzyme thiols-disulfides in metabolic regulation. Annu. Rev. Biochem. 54, 305–329 2862840 10.1146/annurev.bi.54.070185.001513

[8] Sies H. Jones D. P. (eds) (2007) Encyclopedia of Stress: Oxidative Stress, Vol. 3, Academic Press, London

[9] Sies H. (ed) (1985) Oxidative stress: introductory remarks. Oxidative Stress, pp. 1–8, Academic Press, London

[10] McCord J. M. Fridovich I. (1969) Superoxide dismutase. An enzymic function for erythrocuprein (hemocuprein). J. Biol. Chem. 244, 6049–6055 5389100

[11] Suh Y. A. Arnold R. S. Lassegue B. Shi J. Xu X. Sorescu D. Chung A. B. Griendling K. K. Lambeth J. D. (1999) Cell transformation by the superoxide-generating oxidase Mox1. Nature 401, 79–82 10485709 10.1038/43459

[12] Higdon A. Diers A. R. Oh J. Y. Landar A. Darley-Usmar V. M. (2012) Cell signalling by reactive lipid species: new concepts and molecular mechanisms. Biochem. J. 442, 453–464 22364280 10.1042/BJ20111752 PMC3286857

[13] Stadtman E. R. Levine R. L. (2003) Free radical-mediated oxidation of free amino acids and amino acid residues in proteins. Amino Acids 25, 207–218 14661084 10.1007/s00726-003-0011-2

[14] Jones D. P. Park Y. Ziegler T. R. (2012) Nutritional metabolomics: progress in addressing complexity in diet and health. Annu. Rev. Nutr. 32, 183–202 22540256 10.1146/annurev-nutr-072610-145159 PMC4031100

[15] Jones D. P. (2006) Disruption of mitochondrial redox circuitry in oxidative stress. Chem. Biol. Interact. 163, 38–53 16970935 10.1016/j.cbi.2006.07.008

[16] Dooley C. T. Dore T. M. Hanson G. T. Jackson W. C. Remington S. J. Tsien R. Y. (2004) Imaging dynamic redox changes in mammalian cells with green fluorescent protein indicators. J. Biol. Chem. 279, 22284–22293 14985369 10.1074/jbc.M312847200

[17] Jones D. P. (2008) Radical-free biology of oxidative stress. Am. J. Physiol. Cell Physiol. 295, C849–C868 18684987 10.1152/ajpcell.00283.2008 PMC2575825

[18] Jones D. P. Carlson J. L. Mody V. C. Cai J. Lynn M. J. Sternberg P. (2000) Redox state of glutathione in human plasma. Free Radic. Biol. Med. 28, 625–635 10719244 10.1016/s0891-5849(99)00275-0

[19] Gilbert H. F. (1990) Molecular and cellular aspects of thiol-disulfide exchange. Adv. Enzymol. Relat. Areas Mol. Biol. 63, 69–172 2407068 10.1002/9780470123096.ch2

[20] Watson W. H. Pohl J. Montfort W. R. Stuchlik O. Reed M. S. Powis G. Jones D. P. (2003) Redox potential of human thioredoxin 1 and identification of a second dithiol/disulfide motif. J. Biol. Chem. 278, 33408–33415 12816947 10.1074/jbc.M211107200

[21] Gladyshev V. N. Hatfield D. L. (1999) Selenocysteine-containing proteins in mammals. J. Biomed. Sci. 6, 151–160 10343164 10.1007/BF02255899

[22] Brosnan J. T. Brosnan M. E. (2006) The sulfur-containing amino acids: an overview. J. Nutr. 136, 1636S–1640S 16702333 10.1093/jn/136.6.1636S

[23] Stadtman E. R. Van Remmen H. Richardson A. Wehr N. B. Levine R. L. (2005) Methionine oxidation and aging. Biochim. Biophys. Acta 1703, 135–140 15680221 10.1016/j.bbapap.2004.08.010

[24] Moskovitz J. Poston J. M. Berlett B. S. Nosworthy N. J. Szczepanowski R. Stadtman E. R. (2000) Identification and characterization of a putative active site for peptide methionine sulfoxide reductase (MsrA) and its substrate stereospecificity. J. Biol. Chem. 275, 14167–14172 10799493 10.1074/jbc.275.19.14167

[25] Ruan H. Tang X. D. Chen M. L. Joiner M. L. Sun G. Brot N. Weissbach H. Heinemann S. H. Iverson L. Wu C. F. Hoshi T. (2002) High-quality life extension by the enzyme peptide methionine sulfoxide reductase. Proc. Natl. Acad. Sci. U.S.A. 99, 2748–2753 11867705 10.1073/pnas.032671199 PMC122419

[26] Epstein C. J. Goldberger R. F. Anfinsen C. B. (1963) The genetic control of tertiary protein structure: studies with model systems. Cold Spring Harbor Symp. Quant. Biol. 28, 439–449

[27] Bulleid N. J. Ellgaard L. (2011) Multiple ways to make disulfides. Trends Biochem. Sci. 36, 485–492 21778060 10.1016/j.tibs.2011.05.004

[28] Hart P. J. Balbirnie M. M. Ogihara N. L. Nersissian A. M. Weiss M. S. Valentine J. S. Eisenberg D. (1999) A structure-based mechanism for copper-zinc superoxide dismutase. Biochemistry 38, 2167–2178 10026301 10.1021/bi982284u

[29] Zangger K. Armitage I. M. (2002) Dynamics of interdomain and intermolecular interactions in mammalian metallothioneins. J. Inorg. Biochem. 88, 135–143 11803034 10.1016/s0162-0134(01)00379-8

[30] Schafer F. Q. Buettner G. R. (2001) Redox environment of the cell as viewed through the redox state of the glutathione disulfide/glutathione couple. Free Radic. Biol. Med. 30, 1191–1212 11368918 10.1016/s0891-5849(01)00480-4

[31] Billiet L. Geerlings P. Messens J. Roos G. (2012) The thermodynamics of thiol sulfenylation. Free Radic. Biol. Med. 52, 1473–1485 22326773 10.1016/j.freeradbiomed.2011.12.029

[32] Mieyal J. J. Gallogly M. M. Qanungo S. Sabens E. A. Shelton M. D. (2008) Molecular mechanisms and clinical implications of reversible protein *S*-glutathionylation. Antioxid. Redox Signal. 10, 1941–1988 18774901 10.1089/ars.2008.2089 PMC2774718

[33] Casagrande S. Bonetto V. Fratelli M. Gianazza E. Eberini I. Massignan T. Salmona M. Chang G. Holmgren A. Ghezzi P. (2002) Glutathionylation of human thioredoxin: a possible crosstalk between the glutathione and thioredoxin systems. Proc. Natl. Acad. Sci. U.S.A. 99, 9745–9749 12119401 10.1073/pnas.152168599 PMC125000

[34] Hashemy S. I. Holmgren A. (2008) Regulation of the catalytic activity and structure of human thioredoxin 1 via oxidation and *S*-nitrosylation of cysteine residues. J. Biol. Chem. 283, 21890–21898 18544525 10.1074/jbc.M801047200

[35] Ishima Y. Sawa T. Kragh-Hansen U. Miyamoto Y. Matsushita S. Akaike T. Otagiri M. (2007) *S*-Nitrosylation of human variant albumin Liprizzi (R410C) confers potent antibacterial and cytoprotective properties. J. Pharmacol. Exp. Ther. 320, 969–977 17135341 10.1124/jpet.106.114959

[36] Mallis R. J. Buss J. E. Thomas J. A. (2001) Oxidative modification of H-ras: *S*-thiolation and *S*-nitrosylation of reactive cysteines. Biochem. J. 355, 145–153 11256959 10.1042/0264-6021:3550145 PMC1221722

[37] Reynaert N. L. Ckless K. Guala A. S. Wouters E. F. van der Vliet A. Janssen-Heininger Y. M. (2006) *In situ* detection of *S*-glutathionylated proteins following glutaredoxin-1 catalyzed cysteine derivatization. Biochim. Biophys. Acta 1760, 380–387 16515838 10.1016/j.bbagen.2006.01.006

[38] Anand P. Stamler J. S. (2012) Enzymatic mechanisms regulating protein *S*-nitrosylation: implications in health and disease. J. Mol. Med. 90, 233–244 22361849 10.1007/s00109-012-0878-z PMC3379879

[39] Martínez-Ruiz A. Lamas S. (2004) *S*-Nitrosylation: a potential new paradigm in signal transduction. Cardiovasc. Res. 62, 43–52 15023551 10.1016/j.cardiores.2004.01.013

[40] Gadalla M. M. Snyder S. H. (2010) Hydrogen sulfide as a gasotransmitter. J. Neurochem. 113, 14–26 20067586 10.1111/j.1471-4159.2010.06580.x PMC2965526

[41] Mustafa A. K. Gadalla M. M. Sen N. Kim S. Mu W. Gazi S. K. Barrow R. K. Yang G. Wang R. Snyder S. H. (2009) H_2_S signals through protein *S*-sulfhydration. Sci. Signal. 2, ra72 19903941 10.1126/scisignal.2000464 PMC2998899

[42] Li L. Rose P. Moore P. K. (2011) Hydrogen sulfide and cell signaling. Annu. Rev. Pharmacol. Toxicol. 51, 169–187 21210746 10.1146/annurev-pharmtox-010510-100505

[43] Leonard S. E. Reddie K. G. Carroll K. S. (2009) Mining the thiol proteome for sulfenic acid modifications reveals new targets for oxidation in cells. ACS Chem. Biol. 4, 783–799 19645509 10.1021/cb900105q

[44] Rappaport S. M. Li H. Grigoryan H. Funk W. E. Williams E. R. (2012) Adductomics: characterizing exposures to reactive electrophiles. Toxicol. Lett. 213, 83–90 21501670 10.1016/j.toxlet.2011.04.002 PMC4758449

[45] van Montfort R. L. Congreve M. Tisi D. Carr R. Jhoti H. (2003) Oxidation state of the active-site cysteine in protein tyrosine phosphatase 1B. Nature 423, 773–777 12802339 10.1038/nature01681

[46] Kemp M. Go Y. M. Jones D. P. (2008) Nonequilibrium thermodynamics of thiol/disulfide redox systems: a perspective on redox systems biology. Free Radic. Biol. Med. 44, 921–937 18155672 10.1016/j.freeradbiomed.2007.11.008 PMC2587159

[47] Toledano M. B. Leonard W. J. (1991) Modulation of transcription factor NF-κB binding activity by oxidation-reduction *in vitro*. Proc. Natl. Acad. Sci. U.S.A. 88, 4328–4332 1903539 10.1073/pnas.88.10.4328 PMC51652

[48] Robinson L. J. Michel T. (1995) Mutagenesis of palmitoylation sites in endothelial nitric oxide synthase identifies a novel motif for dual acylation and subcellular targeting. Proc. Natl. Acad. Sci. U.S.A. 92, 11776–11780 8524847 10.1073/pnas.92.25.11776 PMC40485

[49] Liu J. García-Cardeña G. Sessa W. C. (1996) Palmitoylation of endothelial nitric oxide synthase is necessary for optimal stimulated release of nitric oxide: implications for caveolae localization. Biochemistry 35, 13277–13281 8873592 10.1021/bi961720e

[50] Anelli T. Alessio M. Bachi A. Bergamelli L. Bertoli G. Camerini S. Mezghrani A. Ruffato E. Simmen T. Sitia R. (2003) Thiol-mediated protein retention in the endoplasmic reticulum: the role of ERp44. EMBO J. 22, 5015–5022 14517240 10.1093/emboj/cdg491 PMC204474

[51] Kondo N. Ishii Y. Son A. Sakakura-Nishiyama J. Kwon Y. W. Tanito M. Nishinaka Y. Matsuo Y. Nakayama T. Taniguchi M. Yodoi J. (2004) Cysteine-dependent immune regulation by TRX and MIF/GIF family proteins. Immunol. Lett. 92, 143–147 15081538 10.1016/j.imlet.2003.11.030

[52] Watarai H. Nozawa R. Tokunaga A. Yuyama N. Tomas M. Hinohara A. Ishizaka K. Ishii Y. (2000) Posttranslational modification of the glycosylation inhibiting factor (GIF) gene product generates bioactive GIF. Proc. Natl. Acad. Sci. U.S.A. 97, 13251–13256 11069294 10.1073/pnas.230445397 PMC27211

[53] Swope M. D. Sun H. W. Klockow B. Blake P. Lolis E. (1998) Macrophage migration inhibitory factor interactions with glutathione and *S*-hexylglutathione. J. Biol. Chem. 273, 14877–14884 9614090 10.1074/jbc.273.24.14877

[54] Mieyal J. J. Chock P. B. (2012) Posttranslational modification of cysteine in redox signaling and oxidative stress: focus on *S*-glutathionylation. Antioxid. Redox Signal. 16, 471–475 22136616 10.1089/ars.2011.4454 PMC3270050

[55] Hansen J. M. Watson W. H. Jones D. P. (2004) Compartmentation of Nrf-2 redox control: regulation of cytoplasmic activation by glutathione and DNA binding by thioredoxin-1. Toxicol. Sci. 82, 308–317 15282410 10.1093/toxsci/kfh231

[56] Kirlin W. G. Cai J. Thompson S. A. Diaz D. Kavanagh T. J. Jones D. P. (1999) Glutathione redox potential in response to differentiation and enzyme inducers. Free Radic. Biol. Med. 27, 1208–1218 10641713 10.1016/s0891-5849(99)00145-8

[57] Jones D. P. (2010) Redox sensing: orthogonal control in cell cycle and apoptosis signalling. J. Intern. Med. 268, 432–448 20964735 10.1111/j.1365-2796.2010.02268.x PMC2963474

[58] Miseta A. Csutora P. (2000) Relationship between the occurrence of cysteine in proteins and the complexity of organisms. Mol. Biol. Evol. 17, 1232–1239 10908643 10.1093/oxfordjournals.molbev.a026406

[59] Go Y. M. Duong D. M. Peng J. Jones D. P. (2011) Protein cysteines map to functional networks according to steady-state level of oxidation. J. Proteomics Bioinform. 4, 196–209 22605892 10.4172/jpb.1000190 PMC3352318

[60] Leichert L. I. Gehrke F. Gudiseva H. V. Blackwell T. Ilbert M. Walker A. K. Strahler J. R. Andrews P. C. Jakob U. (2008) Quantifying changes in the thiol redox proteome upon oxidative stress *in vivo*. Proc. Natl. Acad. Sci. U.S.A. 105, 8197–8202 18287020 10.1073/pnas.0707723105 PMC2448814

[61] Sethuraman M. McComb M. E. Huang H. Huang S. Heibeck T. Costello C. E. Cohen R. A. (2004) Isotope-coded affinity tag (ICAT) approach to redox proteomics: identification and quantitation of oxidant-sensitive cysteine thiols in complex protein mixtures. J. Proteome Res. 3, 1228–1233 15595732 10.1021/pr049887e

[62] Truong T. H. Garcia F. J. Seo Y. H. Carroll K. S. (2011) Isotope-coded chemical reporter and acid-cleavable affinity reagents for monitoring protein sulfenic acids. Bioorg. Med. Chem. Lett. 21, 5015–5020 21601453 10.1016/j.bmcl.2011.04.115

[63] Murphy M. P. (2012) Mitochondrial thiols in antioxidant protection and redox signaling: distinct roles for glutathionylation and other thiol modifications. Antioxid. Redox Signal. 16, 476–495 21954972 10.1089/ars.2011.4289

[64] Go Y. M. Pohl J. Jones D. P. (2009) Quantification of redox conditions in the nucleus. Methods Mol. Biol. 464, 303–317 18951192 10.1007/978-1-60327-461-6_17

[65] Kim J. R. Lee S. M. Cho S. H. Kim J. H. Kim B. H. Kwon J. Choi C. Y. Kim Y. D. Lee S. R. (2004) Oxidation of thioredoxin reductase in HeLa cells stimulated with tumor necrosis factor-α. FEBS Lett. 567, 189–196 15178321 10.1016/j.febslet.2004.04.055

[66] Kim J. R. Yoon H. W. Kwon K. S. Lee S. R. Rhee S. G. (2000) Identification of proteins containing cysteine residues that are sensitive to oxidation by hydrogen peroxide at neutral pH. Anal. Biochem. 283, 214–221 10906242 10.1006/abio.2000.4623

[67] Skalska J. Brookes P. S. Nadtochiy S. M. Hilchey S. P. Jordan C. T. Guzman M. L. Maggirwar S. B. Briehl M. M. Bernstein S. H. (2009) Modulation of cell surface protein free thiols: a potential novel mechanism of action of the sesquiterpene lactone parthenolide. PLoS ONE 4, e8115 19956548 10.1371/journal.pone.0008115 PMC2780735

[68] Fratelli M. Demol H. Puype M. Casagrande S. Eberini I. Salmona M. Bonetto V. Mengozzi M. Duffieux F. Miclet E. Bachi A. Vandekerckhove J. Gianazza E. Ghezzi P. (2002) Identification by redox proteomics of glutathionylated proteins in oxidatively stressed human T lymphocytes. Proc. Natl. Acad. Sci. U.S.A. 99, 3505–3510 11904414 10.1073/pnas.052592699 PMC122553

[69] Le Moan N. Clement G. Le Maout S. Tacnet F. Toledano M. B. (2006) The *Saccharomyces cerevisiae* proteome of oxidized protein thiols: contrasted functions for the thioredoxin and glutathione pathways. J. Biol. Chem. 281, 10420–10430 16418165 10.1074/jbc.M513346200

[70] Butterfield D. A. Perluigi M. Sultana R. (2006) Oxidative stress in Alzheimer's disease brain: new insights from redox proteomics. Eur. J. Pharmacol. 545, 39–50 16860790 10.1016/j.ejphar.2006.06.026

[71] Go Y. M. Jones D. P. (2013) Thiol/disulfide redox states in signaling and sensing. Crit. Rev. Biochem. Mol. Biol. 48, 173–181 23356510 10.3109/10409238.2013.764840 PMC4323379

[72] Jones D. P. (2002) Redox potential of GSH/GSSG couple: assay and biological significance. Methods Enzymol. 348, 93–112 11885298 10.1016/s0076-6879(02)48630-2

[73] Nagy P. Winterbourn C. C. (2010) Redox chemistry of biological thiols. Adv. Mol. Toxicol. 4, 183–222

[74] Hegde P. S. White I. R. Debouck C. (2003) Interplay of transcriptomics and proteomics. Curr. Opin. Biotechnol. 14, 647–651 14662396 10.1016/j.copbio.2003.10.006

[75] D'Autréaux B. Toledano M. B. (2007) ROS as signalling molecules: mechanisms that generate specificity in ROS homeostasis. Nat. Rev. Mol. Cell Biol. 8, 813–824 17848967 10.1038/nrm2256

[76] Mesecke N. Terziyska N. Kozany C. Baumann F. Neupert W. Hell K. Herrmann J. M. (2005) A disulfide relay system in the intermembrane space of mitochondria that mediates protein import. Cell 121, 1059–1069 15989955 10.1016/j.cell.2005.04.011

[77] Banerjee R. (2012) Redox outside the box: linking extracellular redox remodeling with intracellular redox metabolism. J. Biol. Chem. 287, 4397–4402 22147695 10.1074/jbc.R111.287995 PMC3281658

[78] Wang J. Boja E. S. Tan W. Tekle E. Fales H. M. English S. Mieyal J. J. Chock P. B. (2001) Reversible glutathionylation regulates actin polymerization in A431 cells. J. Biol. Chem. 276, 47763–47766 11684673 10.1074/jbc.C100415200

[79] Appenzeller-Herzog C. Ellgaard L. (2008) *In vivo* reduction-oxidation state of protein disulfide isomerase: the two active sites independently occur in the reduced and oxidized forms. Antioxid. Redox Signal. 10, 55–64 17939758 10.1089/ars.2007.1837

[80] Knoefler D. Thamsen M. Koniczek M. Niemuth N. J. Diederich A. K. Jakob U. (2012) Quantitative *in vivo* redox sensors uncover oxidative stress as an early event in life. Mol. Cell 47, 767–776 22819323 10.1016/j.molcel.2012.06.016 PMC3444654

[81] Go Y. M. Park H. Koval M. Orr M. Reed M. Liang Y. Smith D. Pohl J. Jones D. P. (2010) A key role for mitochondria in endothelial signaling by plasma cysteine/cystine redox potential. Free Radic. Biol. Med. 48, 275–283 19879942 10.1016/j.freeradbiomed.2009.10.050 PMC3057402

[82] Shartava A. Korn W. Shah A. K. Goodman S. R. (1997) Irreversibly sickled cell β-actin: defective filament formation. Am. J. Hematol. 55, 97–103 9209005 10.1002/(sici)1096-8652(199706)55:2<97::aid-ajh8>3.0.co;2-y

[83] Caprari P. Bozzi A. Malorni W. Bottini A. Iosi F. Santini M. T. Salvati A. M. (1995) Junctional sites of erythrocyte skeletal proteins are specific targets of *tert*-butylhydroperoxide oxidative damage. Chem. Biol. Interact. 94, 243–258 7820887 10.1016/0009-2797(94)03339-a

[84] Laragione T. Bonetto V. Casoni F. Massignan T. Bianchi G. Gianazza E. Ghezzi P. (2003) Redox regulation of surface protein thiols: identification of integrin α-4 as a molecular target by using redox proteomics. Proc. Natl. Acad. Sci. U.S.A. 100, 14737–14741 14657342 10.1073/pnas.2434516100 PMC299788

[85] Fiaschi T. Cozzi G. Raugei G. Formigli L. Ramponi G. Chiarugi P. (2006) Redox regulation of β-actin during integrin-mediated cell adhesion. J. Biol. Chem. 281, 22983–22991 16757472 10.1074/jbc.M603040200

[86] Wang X. Ling S. Zhao D. Sun Q. Li Q. Wu F. Nie J. Qu L. Wang B. Shen X. Bai Y. Li Y. (2010) Redox regulation of actin by thioredoxin-1 is mediated by the interaction of the proteins via cysteine 62. Antioxid. Redox Signal. 13, 565–573 20218863 10.1089/ars.2009.2833

[87] Thom S. R. Bhopale V. M. Milovanova T. N. Yang M. Bogush M. (2012) Thioredoxin reductase linked to cytoskeleton by focal adhesion kinase reverses actin *S*-nitrosylation and restores neutrophil β_2_ integrin function. J. Biol. Chem. 287, 30346–30357 22778269 10.1074/jbc.M112.355875 PMC3436286

[88] Salzano A. M. Paron I. Pines A. Bachi A. Talamo F. Bivi N. Vascotto C. Damante G. Quadrifoglio F. Scaloni A. Tell G. (2006) Differential proteomic analysis of nuclear extracts from thyroid cell lines. J. Chromatogr. B Analyt. Technol. Biomed. Life Sci. 833, 41–50 10.1016/j.jchromb.2005.12.025 16431169

[89] Biteau B. Labarre J. Toledano M. B. (2003) ATP-dependent reduction of cysteine-sulphinic acid by *S. cerevisiae* sulphiredoxin. Nature 425, 980–984 14586471 10.1038/nature02075

[90] Jeong W. Park S. J. Chang T. S. Lee D. Y. Rhee S. G. (2006) Molecular mechanism of the reduction of cysteine sulfinic acid of peroxiredoxin to cysteine by mammalian sulfiredoxin. J. Biol. Chem. 281, 14400–14407 16565085 10.1074/jbc.M511082200

[91] Findlay V. J. Townsend D. M. Morris T. E. Fraser J. P. He L. Tew K. D. (2006) A novel role for human sulfiredoxin in the reversal of glutathionylation. Cancer Res. 66, 6800–6806 16818657 10.1158/0008-5472.CAN-06-0484 PMC6361143

[92] Morot-Gaudry-Talarmain Y. (2009) Physical and functional interactions of cyclophilin B with neuronal actin and peroxiredoxin-1 are modified by oxidative stress. Free Radic. Biol. Med. 47, 1715–1730 19766713 10.1016/j.freeradbiomed.2009.09.014

[93] Kuiper J. W. Sun C. Magalhães M. A. Glogauer M. (2011) Rac regulates PtdInsP_3_ signaling and the chemotactic compass through a redox-mediated feedback loop. Blood 118, 6164–6171 21976675 10.1182/blood-2010-09-310383

[94] Vandermoere F. El Yazidi-Belkoura I. Demont Y. Slomianny C. Antol J. Lemoine J. Hondermarck H. (2007) Proteomics exploration reveals that actin is a signaling target of the kinase Akt. Mol. Cell. Proteomics 6, 114–124 17088265 10.1074/mcp.M600335-MCP200

[95] Sies H. (ed) (1982) Metabolic Compartmentation, Academic Press, London

[96] Yu T. Park Y. Johnson J. M. Jones D. P. (2009) apLCMS–adaptive processing of high-resolution LC/MS data. Bioinformatics 25, 1930–1936 19414529 10.1093/bioinformatics/btp291 PMC2712336

[97] Uppal K. Soltow Q. A. Strobel F. H. Pittard W. S. Gernert K. M. Yu T. Jones D. P. (2013) xMSanalyzer: automated pipeline for improved feature detection and downstream analysis of large-scale, non-targeted metabolomics data. BMC Bioinformatics 14, 15 23323971 10.1186/1471-2105-14-15 PMC3562220

[98] Roede J. R. Park Y. Li S. Strobel F. H. Jones D. P. (2012) Detailed mitochondrial phenotyping by high resolution metabolomics. PLoS ONE 7, e33020 22412977 10.1371/journal.pone.0033020 PMC3295783

